# Umbilical Cord as Prospective Source for Mesenchymal Stem Cell-Based Therapy

**DOI:** 10.1155/2016/6901286

**Published:** 2016-08-29

**Authors:** Irina Arutyunyan, Andrey Elchaninov, Andrey Makarov, Timur Fatkhudinov

**Affiliations:** ^1^Research Center for Obstetrics, Gynecology and Perinatology, Ministry of Healthcare of the Russian Federation, No. 4, Oparin Street, Moscow 117997, Russia; ^2^Pirogov Russian National Research Medical University, Ministry of Healthcare of the Russian Federation, No. 1, Ostrovitianov Street, Moscow 117997, Russia

## Abstract

The paper presents current evidence on the properties of human umbilical cord-derived mesenchymal stem cells, including origin, proliferative potential, plasticity, stability of karyotype and phenotype, transcriptome, secretome, and immunomodulatory activity. A review of preclinical studies and clinical trials using this cell type is performed. Prospects for the use of mesenchymal stem cells, derived from the umbilical cord, in cell transplantation are associated with the need for specialized biobanking and transplant standardization criteria.

## 1. Introduction

Many researchers consider the transplantation of mesenchymal stem cells (MSCs) to be the most effective tool for cell therapy, due to the simultaneous activation of multiple mechanisms (paracrine, trophic, immunomodulatory, and differentiation), affecting all stages of the regeneration of damaged tissues. Bone marrow-derived MSCs (BM-MSCs) are the most extensively characterized as they are the historically accepted “gold standard” of MSCs. Nevertheless, currently there is active research work regarding MSCs from other sources—adipose tissue, peripheral and umbilical cord blood, amniotic fluid, skin, dental pulp, synovium, umbilical cord tissue, placental complex, endometrium, and others. In fact, evidence has suggested that MSCs may be present virtually in any vascularized tissue throughout the whole body [1]. All these cell types meet the minimum criteria for MSCs but have significant differences in their features. Our review focuses on umbilical cord-derived MSCs (UC-MSCs), cells that have a unique combination of prenatal and postnatal stem cell properties.

## 2. The Origin and Morphology of the Human Umbilical Cord

**Figure 1:**
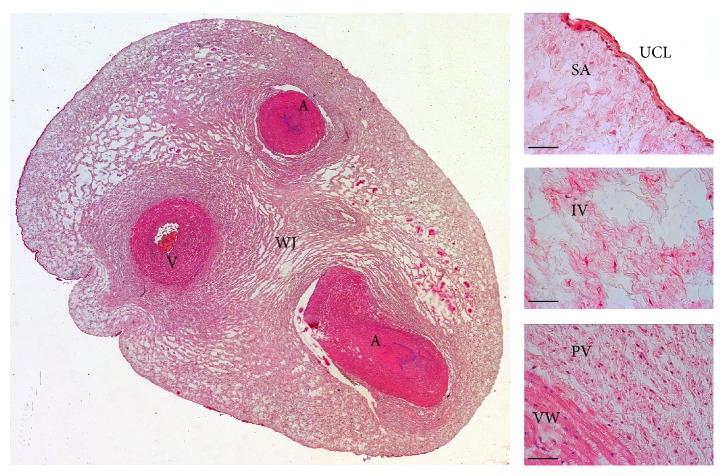
Cross-section of the human umbilical cord. A: artery; V: vein; WJ: Wharton's jelly; UCL: umbilical cord lining; SA, IV, and PV: subamnion, intervascular, and perivascular zones of Wharton's jelly; VW: blood vessel wall. Hematoxylin and eosin staining, scale bar = 200 *µ*m.

The umbilical cord develops from the yolk sac and allantois and becomes a conduit between the developing embryo or fetus and the placenta. The umbilical cord stroma contains gelatinous substance called Wharton's jelly after Thomas Wharton (1614–1673), an English physician and anatomist. Wharton's jelly protects the blood vessels (two umbilical arteries and one umbilical vein) from clumping and provides cord flexibility. This substance is made largely from glycosaminoglycans, especially hyaluronic acid and chondroitin sulfate. Collagen fibers are the main fibrillary component, while elastic fibers are absent. The cell component is presented by mesenchyme-derived cells (fibroblasts, myofibroblasts, smooth muscle cells, and mesenchymal stem cells) [2]. In contrast to most tissues of the body, there are no capillaries in Wharton's jelly: there is an active process of hematopoiesis and capillaries formation in umbilical cord stroma at week 6 of development; however, at 7–9 weeks, hematopoiesis stops and capillaries regress [3]. Cross-section of the human umbilical cord is shown in Figure 1.

## 3. The Umbilical Cord as a Source of Mesenchymal Stem Cells

In 1974, umbilical cord blood was declared to be the source of hematopoietic stem and progenitor cells [4], and the remaining umbilical cord tissue was considered medical waste with no scientific value. This point of view was completely revised in 1991, when McElreavey et al. isolated fibroblast-like cells from Wharton's jelly and characterized them [5]. In 2004, these fibroblast-like cells were proved to be MSCs as they expressed CD29, CD44, CD51, CD73, and CD105, lacked expression of CD34 and CD45, and were able to differentiate into cells of the adipogenic and osteogenic lineages [6]. Currently, the umbilical cord MSCs include cells derived from the total umbilical cord or its different sections (perivascular, intervascular, and subamnion zones of Wharton's jelly and subendothelial layer but not from umbilical cord lining or inner blood vessel walls) [2].

**Figure 2:**
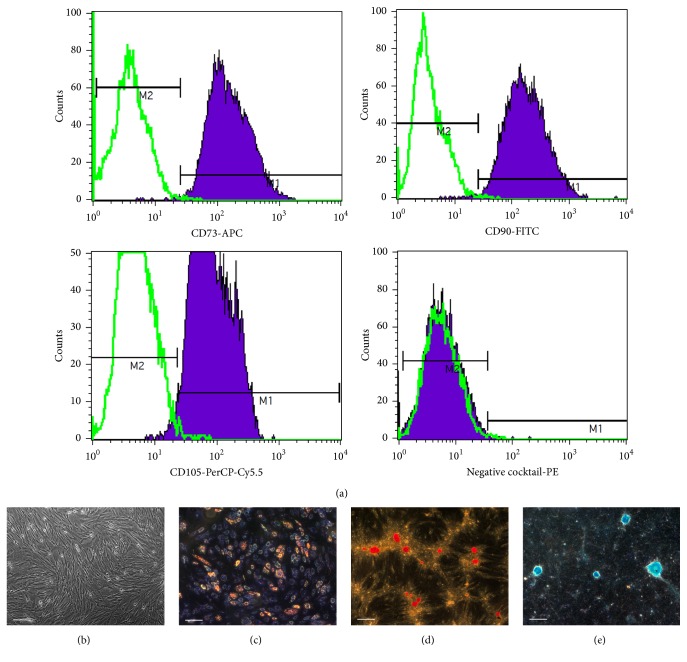
The characteristics of cultured cells derived from Wharton's jelly according to minimal criteria to define human MSCs proposed by ISCT. (a) Analysis of immunophenotype with BD Stemflow*™* hMSC Analysis Kit (BD Biosciences). Negative MSC cocktail includes PE CD45, PE CD34, PE CD11b, PE CD19, and PE HLA-DR antibody conjugates. (b) Phase contrast capture of UC-MSCs at the fourth passage. Scale bar: 200 *μ*m. (c) Adipogenic differentiation with StemPro® Adipogenesis Differentiation Kit (Gibco). Lipid droplets are stained with Sudan III. Scale bar: 200 *μ*m. (d) Osteogenic differentiation with StemPro Osteogenesis Differentiation Kit (Gibco). Calcificated nodules are stained with Alizarin red S (pH = 4.1). Scale bar: 200 *μ*m. (e) Chondrogenic differentiation with StemPro Chondrogenesis Differentiation Kit (Gibco). Mucopolysaccharides are stained with Alcian blue (pH = 2.5). Scale bar: 200 *μ*m.

Figure 2 shows the characteristics of cultured cells derived from Wharton's jelly according to the minimal criteria to define human MSCs as proposed by the Mesenchymal and Tissue Stem Cell Committee of the International Society for Cellular Therapy (ISCT): (1) MSCs must be plastic-adherent when maintained in standard culture conditions; (2) MSCs must express CD105, CD73, and CD90 and lack expression of CD45, CD34, CD14 or CD11b, CD79a or CD19, and HLA-DR surface molecules; (3) MSCs must differentiate into osteoblasts, adipocytes, and chondroblasts* in vitro* [40].

## 4. The Origin of Wharton's Jelly MSCs

In 2008, Wang et al. presumed that early in embryogenesis, hematopoietic cells and MSCs migrate from the yolk sac and aorta-gonad-mesonephros to the placenta and then back to the fetal liver and bone marrow through the umbilical cord. During these two migration waves, some cells are trapped in Wharton's jelly and are retained therein throughout the whole period of gestation. The new microenvironment changes the properties of migrating cells, which probably explains their differences from BM-MSCs [41].

## 5. Isolation of Primary Cell Culture from Wharton's Jelly

Most protocols for primary cell culture isolation from Wharton's jelly consist of three steps:

1. Removal of the epithelial, vascular, and perivascular tissues.
2. Mechanical grinding and enzymatic digestion using trypsin, collagenases I, II, or IV, dispase, protease, and hyaluronidase.
3. Transfer into the culture medium (standard culture media with human or fetal calf serum which may be supplemented with growth factors FGFb, EGF, PDGF, and VEGF) [2, 7, 42].

In addition, an explant culture method can be applied; it avoids the damaging effects of enzymes on cells and reduces the processing time of the biomaterial (“plate and wait” procedure) [43]. The common explant method of isolating UC-MSCs involves mincing the umbilical cords into small fragments, which are then attached to a culture dish bottom from which the cells migrate. One of the disadvantages of this method is that the fragments frequently float up from the bottom of the dish, thereby reducing the cell recovery rate. In some protocols, a stainless steel mesh is used to protect the tissue from floating [44].

According to some reports, the explant method allows the selection of a cell fraction with higher proliferative potential [45, 46], but a remarkable variation of cell phenotype expressions was distinguished compared to enzymatic digestion [47, 48]. In a recent study, three explant culture methods and three enzymatic methods were compared. MSC isolation using the 10 mm size tissue explant method led to shorter primary culture time, higher numbers of isolated cells, and higher proliferation rates compared with other isolation methods. Immune phenotype and multilineage differentiation capacity did not differ significantly among six groups [49]. It was also found that UC-MSCs isolated by explant technique always reached proliferation arrest earlier, irrespective of initial population doubling times, but the mechanism explaining this effect is still unclear [50]. On the contrary, later studies showed that cells obtained from explants presented similar characteristics (morphology, population doubling time, postthaw survival, differentiation capacity, and phenotype) to those from enzymatic protocols [51].

According to published data [52, 53] and our own laboratory data, the efficacy of isolation of primary cell culture from Wharton's jelly amounts to 100%. In comparison, the efficacy of MSC isolation from umbilical cord blood does not exceed 60%; amniotic fluid is 90%; placenta varies from 62.5% to 100% [52, 53]. Wharton's jelly tissue yields the highest concentration of allogeneic mesenchymal stem cells: yields for bone marrow ranged from 1 to 317,400 cells/mL; yields for adipose tissue ranged from 4,737 to 1,550,000 cells/mL of tissue; and yields for umbilical cord tissue ranged from 10,000 to 4,700,000 cells/cm of umbilical cord [54].

It should be particularly noted that almost all culture laboratories use umbilical cords obtained after Caesarean sections, because vaginal delivery significantly increases the risk of contamination of primary biological material. Some researchers suppose that viable MSCs can only be isolated from fresh umbilical cord tissue, not from frozen tissue fragments [55]. According to another report, MSCs derived from frozen cord tissue exhibited decreased plating efficiency and increased doubling times but near equivalent maximum cell expansion compared with fresh cord tissue [56].

## 6. The Proliferative Potential and the Karyotype Stability of UC-MSCs

UC-MSCs have higher proliferative potential than BM-MSCs (the “gold standard” for MSCs comparison) or MSCs from other postnatal (adipose tissue) and neonatal sources (placenta and amniotic membrane) [13, 16, 17, 57, 58]. The mean of CFU-F (colony-forming unit-fibroblast) colonies per 1 × 10^6^ nucleated cells was significantly higher in UC-MSCs (800, range 300–2000) than in BM-MSCs (36, range 16–64) as determined by the CFU-F assay based on Castro-Malaspina's method [57]. CFU-F frequency determined by limiting dilution assay also confirmed a higher frequency of CFU-F in UC-MSCs (1 : 1609 ± 0.18) than in BM-MSCs (1 : 35700 ± 0.01) [57]. According to another report, typical CFU-F efficiency (the ratio of number of cells forming colonies under clonal conditions and number of cells seeded directly after isolation) for BM-MSCs ranged from 0.001% to 0.01%, while for UC-MSCs it reached 0.2–1.8% [59].

It has been reported that cell doubling time for UC-MSCs approximates 21 h [58], 24 h [57], 40 h [13, 46], and 45 h [56]. Importantly, according to recent data, each individual UC-MSCs sample exhibited different population doubling rates and reached senescence at different passages due to unique genetic and epigenetic profiles, irrespective of isolation protocol [50]. A sufficient amount of the starting biomaterial (umbilical cord weight is nearly 40 g) and high telomerase activity of UC-MSCs permit obtaining 10^9^ cells from one cord while maintaining their normal karyotype for 6 passages [60, 61]. Since passage 7, the telomerase activity of UC-MSCs is significantly reduced; but cell karyotype is stable for at least 25 passages [22, 62].

## 7. UC-MSCs Phenotype

To date, the expression profile of surface markers and pluripotency markers of UC-MSCs has been investigated extensively (Tables 1 and 2).

Particular attention is drawn to CD105 (endoglin, a part of the TGF beta receptor complex). According to ISCT decision, CD105 is a required marker for MSCs verification [40]; however, different data contradict each other. In most studies, it has been shown that CD105 presents on UC-MSCs surface [2, 7–9], and its expression is maintained during long-term cultivation (at least 16 passages) [63]. However, a few studies have demonstrated that UC-MSCs do not express CD105 at all [64] or until passage 5 [65]. Reduction in mesenchymal-marker (CD73, CD90, and CD105) expression on UC-MSCs may occur under ischemic conditions influenced mainly by hypoxia [66]. In accordance with our laboratory data, more than 98% of the UC-MSCs express CD105 on passages 2–5 as measured by flow cytometry (Figure 2).

Data about the expression of pluripotent specific markers on UC-MSCs are contradictory. In different reports, the expression of these markers was shown only under certain conditions: solely on early passages [67], or when grown in the presence of human embryonic stem cells medium on mouse feeder cells [10], or after lowering O_2_ concentration from 21% to 5% level [68], or after the selection of CD105+ cells and their subsequent cultivation under suspension culture condition [69]. Flow cytometric analysis revealed that neural ganglioside GD2(+)-sorted UC-MSCs showed increased expression of SSEA-4, OCT4, SOX2, and NANOG in comparison to unsorted or GD2-negative cells [70].

## 8. Transcriptomic Profile of UC-MSCs

In 2012, De Kock et al. studied the global gene expression profiles of four human mesoderm-derived stem cell populations. Human UC-MSCs showed significant enrichment in functional gene classes involved in liver and cardiovascular system development and function compared to MSCs derived from adipose tissue, bone marrow, and skin [12]. The most significant differences were found for genes presented in Table 3.

In 2010, Hsieh et al. published interesting data comparing the gene expression profiles of BM-MSCs and UC-MSCs. It was found that, for the two MSC types, there were no common genes among the top 50 known genes most strongly expressed! Top 10 for UC-MSCs included genes encoding somatostatin receptor 1, member 4 of immunoglobulin superfamily, gamma 2 smooth muscle actin, reticulon 1, natriuretic peptide precursor B, keratin 8, desmoglein 2, oxytocin receptor, desmocollin 3, and myocardin. The study also showed that genes related to cell proliferation (*EGF*), PI3K-NFkB signaling pathway (*TEK*), and neurogenesis (*RTN1*,* NPPB*, and* NRP2*) were upregulated in UC-MSCs compared to in BM-MSCs [71].

The UC-MSCs and BM-MSCs were also screened for their surface expression of HLA antigens, costimulatory factors, and immune tolerance molecules [9, 13]. It was found that the expression of MHCII molecules (HLA-DMA, -DRA, and -DPB1) in the BM-MSCs was 16-fold, 36-fold, and 4-fold higher, respectively, compared with the UC-MSCs. The expression levels of immune-related genes* TLR4*,* TLR3*,* JAG1*,* NOTCH2*, and* NOTCH3* in the BM-MSCs were 38-fold, 4-fold, 5-fold, 3-fold, and 4-fold higher, respectively, compared with the UC-MSCs [13]. These results promise successful future use of allogeneic UC-MSCs for clinical trials.

A more detailed comparative analysis of the UC-MSCs transcriptome is presented in the review by El Omar et al. [9].

## 9. The Multilineage Differentiation Potential of UC-MSCs

*In vitro* UC-MSCs showed very high differentiation capacity: these cells were able to differentiate into chondrocytes, adipocytes, osteoblasts, odontoblast-like cells, dermal fibroblasts, smooth muscle cells, skeletal muscle cells, cardiomyocytes, hepatocyte-like cells, insulin-producing cells, glucagon-producing cells, and somatostatin-producing cells, sweat gland cells, endothelial cells, neuroglia cells (oligodendrocytes), and dopaminergic neurons [8, 15, 21, 42, 72–75]. In 2014, it was found that under specific conditions UC-MSCs expressed markers of male germ-like cells and primordial-like germ cells; such a possibility had previously been shown only for embryonic stem cells (ESCs) or induced pluripotent stem cells [76, 77].

Comparison of the differentiation potential of UC-MSCs and MSCs from other sources (bone marrow and adipose tissue) is the subject of numerous studies presented in Table 4.

A number of studies have demonstrated the possibility of UC-MSCs' differentiation after genetic modification (transduction or transfection). UC-MSCs overexpressing hepatocyte growth factor (HGF) could differentiate into dopaminergic neuron-like cells secreting dopamine, tyrosine hydroxylase, and dopamine transporter [78] and promoted nerve fiber remyelination and axonal regeneration one week after transplantation in rats with collagenase-induced intracerebral hemorrhage [30]. After infection with adenovirus containing SF-1 cDNA, UC-MSCs had significantly higher expression of all steroidogenic mRNAs (including P450 side-chain cleavage enzyme, 3*β*-HSD, 17*β*-HSD type 3, LH-R, ACTH-R, P450c21, and CYP17), secreted significantly more steroidogenic hormones (including testosterone and cortisol), and had significantly higher cell viability than differentiated BM-MSCs [79].

Interestingly, the plasticity of UC-MSCs may depend on the conditions of pregnancy. UC-MSCs from preeclamptic patients were more committed to neuroglial differentiation: the protein expressions of neuronal (MAP-2) and oligodendrocytic (MBP) markers were significantly increased in cells from preeclampsia versus gestational age-matched controls [80]. At the same time, preterm birth had no effect on neuronal differentiation of UC-MSCs when compared to term delivery [81] but led to a decrease in osteogenic potential [82]. UC-MSCs obtained from gestational diabetes mellitus patients expressed similar levels of CD105, CD90, and CD73 when compared with UC-MSCs from normal pregnant women but showed decreased cell growth and earlier cellular senescence with accumulation of p16 and p53, displayed significantly lower osteogenic and adipogenic differentiation potentials, and, furthermore, exhibited low mitochondrial activity and significantly reduced expression of the mitochondrial function regulatory genes* ND2*,* ND9*,* COX1*,* PGC-1α*, and TFAM [83]. Thus, impaired metabolism of the maternal organism during pregnancy has a significant impact on the biological properties of neonatal MSCs. This fact should be taken into account when choosing a source of cells for clinical use.

## 10. Secretome of UC-MSCs

MSCs produce a variety of bioactive compounds that supply a paracrine mechanism for their therapeutic activity. However, UC-MSCs' secretome differs significantly from MSCs from other sources (bone marrow and adipose tissue). The most obvious dissimilarity is the almost complete absence of synthesis of the main proangiogenic factor VEGF-A: the level of secretion is 10^2^ less than AT-MSCs and 10^3^ less than BM-MSCs [59, 84, 85]. Wherein, transcription level of VEGF gene expression is detectable [85] and, according to some reports, is very similar to that of BM-MSC [57]. The production of some proangiogenic factors (including angiogenin and PLGF) by UC-MSCs is also reduced, and the production of some antiangiogenic factors (including thrombospondin-2 and endostatin) is increased compared with BM-MSCs and AT-MSCs [84, 85]. Contrariwise, UC-MSCs expressed higher levels of angiogenic chemokines such as CXCL1, CXCL, CXCL5, CXCL6, and CXCL8 and angiogenic growth factors like HGF, bFGF, VEGF-D, PDGF-AA, TGF-*β*2, G-CSF, and TGF-*β*2 [59, 84, 86, 87]. Consequently, UC-MSCs realize their proangiogenic capacity by a VEGF-A-independent pathway [20, 85].

It has also been reported that UC-MSCs exhibited increased secretion of neurotrophic factors such as bFGF, nerve growth factor (NGF), neurotrophin 3 (NT3), neurotrophin 4 (NT4), and glial-derived neurotrophic factor (GDNF) compared to BM-MSCs and AT-MSCs [14]. Based on these and published data, the authors of the study believe that UC-MSCs could be precommitted to an ectodermal fate.

Additionally, UC-MSCs secrete significantly higher amounts of several important cytokines and hematopoietic growth factors, including G-CSF, GM-CSF, LIF, IL-1*α*, IL-6, IL-8, and IL-11, compared to BM-MSCs, and thus are better candidates for hematopoietic stem cells expansion [88].

## 11. The Immunomodulatory Properties of UC-MSCs

In 2008, Weiss et al. were the first to investigate the immunomodulatory properties of UC-MSCs.* In vitro* study supported five main conclusions:

1. UC-MSCs suppressed the proliferation of Con-A-stimulated rat splenocytes (xenograft model) or activated human peripheral blood mononuclear cells (allogeneic model).
2. UC-MSCs did not stimulate the proliferation of allogeneic or xenogeneic immune cells.
3. UC-MSCs produced an immunosuppressive isoform of human leukocyte antigen HLA-G6 that inhibited the cytolytic activity of NK cells.
4. UC-MSCs did not express immune response-related surface antigens CD40, CD80, and CD86, which participated in T lymphocytes activation.
5. UC-MSCs produced anti-inflammatory cytokines, which provided their immunomodulatory properties [89].

It is currently believed that the immunomodulatory activity of UC-MSCs is provided by the paracrine mechanism. For example, UC-MSCs produce IL-6 that instructs dendritic cells to acquire tolerogenic phenotypes [90], prostaglandin E2 (PGE2) that suppresses NK cells cytotoxicity [91] and CD4+ and CD8+ T-cell proliferation [92], and indoleamine 2,3-dioxygenase (IDO) that inhibits the differentiation of circulating T follicular helper cells [93]. In contrast to BM-MSCs and AT-MSCs, UC-MSCs secrete anti-inflammatory cytokine IFN-*α* [84]. After exposure with proinflammatory cytokine IL-1*β* for 48 hours, UC-MSCs exhibited comparatively elevated expression of immunomodulatory molecules TGF*β*1, IDO, TNF-stimulated gene 6 protein (TSG-6), and PGE2, when compared to MSCs from bone marrow or placenta [22]. PGE2 secreted by activated MSCs drives resident macrophages with M1 proinflammatory phenotype toward M2 anti-inflammatory phenotype and TSG-6 interacts with CD44 on resident macrophages to decrease TLR2/NF*κ*-B signaling and thereby decrease the secretion of proinflammatory mediators of inflammation. These findings place MSCs (and especially UC-MSCs due to their secretome) at the center of early regulators of inflammation [94].

Interestingly, culture conditions may influence the UC-MSCs' immunomodulatory properties: UC-MSCs-mediated suppression of T-cell proliferation in an allogeneic mixed lymphocyte reaction is more effective in xeno-free (containing GMP-certified human serum) and serum-free media than in standard fetal bovine serum-containing cultures. Therefore, the removal of xenogeneic components of the culture medium is important for future clinical study design in regenerative and transplant medicine [95].

## 12. Mitochondrial Transfer between UC-MSCs and Damaged Cells

About ten years ago the unexpected observation that MSCs can rescue cells with nonfunctional mitochondria by the transfer of either mitochondria or mitochondrial DNA was made [96]. The observation had broad implications for the therapeutic potentials of MSCs because failure of mitochondria is an initial event in many diseases, particularly with ischemia and reperfusion of tissues [97].

In a recent study, the capability of UC-MSCs to transfer their own mitochondria into mitochondrial DNA- (mtDNA-) depleted *ρ*(0) cells was shown. The survival cells had mtDNA identical to that of UC-MSCs, whereas they expressed cellular markers identical to that of *ρ*(0) cells. Importantly, these *ρ*(0)-plus-UC-MSC-mtDNA cells recovered the expression of mtDNA-encoded proteins and exhibited functional oxygen consumption and respiratory control, as well as the activity of electron transport chain (ETC) complexes I, II, III, and IV. In addition, ETC complex V-inhibitor-sensitive ATP production and metabolic shifting were also recovered. Furthermore, cellular behaviors including attachment-free proliferation, aerobic viability, and oxidative phosphorylation-reliant cellular motility were also regained after mitochondrial transfer by UC-MSCs. The therapeutic effect of UC-MSCs-derived mitochondrial transfer was stably sustained for at least 45 passages [98].

The transfer of mitochondria therefore provided a rational for the therapeutic use of UC-MSCs for ischemic injury or diseases linked to mitochondrial dysfunction.

## 13. Tumorigenic Potential of UC-MSCs

Perinatal stem cells possess the characteristics of both embryonic stem cells and adult stem cells as they possess pluripotency properties, as well as multipotent tissue maintenance; they represent a bridge between embryonic and adult stem cells [99]. Expression of markers of pluripotency in the UC-MSCs is higher than in BM-MSCs [8, 11, 72] but lower than in ESCs [41, 100]. Perhaps this explains the crucial difference between UC-MSCs and ESCs: UC-MSCs do not induce tumorigenesis, unlike ESCs. In one of the first works devoted to the subject, the tumor-producing capabilities of UC-MSCs were compared with human ESCs using the immunodeficient mouse model. Animals that received human ESCs developed teratomas in 6 weeks (s.c. 85%; i.m. 75%; i.p. 100%) that contained tissues of ectoderm, mesoderm, and endoderm. No animal that received human UC-MSCs developed tumors or inflammatory reactions at the injection sites when maintained for a prolonged period (20 weeks) [101]. Moreover, it was shown that UC-MSCs could be immortalized by transduction with a lentiviral vector carrying* hTERT* (human telomerase reverse transcriptase) catalytic subunit gene but even then transfected UC-MSCs showed no transformation into tumors in nude mice [102].

*In vitro* model of cell culture transformation (cells were grown in the presence of breast and ovarian cancer cell conditioned medium for 30 days) demonstrated that no changes were observed in UC-MSCs' morphology, proliferation rates, or transcriptome compared to BM-MSCs that transformed into tumor-associated fibroblasts [103].

Therefore, human UC-MSCs, being nontumorigenic, have the potential for safe cell-based therapies.

## 14. Preclinical Studies regarding the Use of UC-MSCs

Promising results were obtained in recent preclinical studies regarding the use of UC-MSCs for the treatment of different diseases using animal models.  Table 5 shows the most interesting data.

Reports from the early period of MSC-based cell therapy for tissue repair demonstrated that injected MSCs may survive, engraft, and differentiate into specific cell types and repair injured tissues. However, subsequent studies supported the notion that the level of UC-MSCs engraftment in the host organs of recipient animals was low after systemic administration and rather high after local administration. There is little evidence for the differentiation of UC-MSCs into relevant cells; it may be related to xenogeneic transplantation used in most of the studies. Presently, proposed mechanisms of UC-MSCs therapeutic activity include trophic and paracrine effects on cells of the immune system, remodeling of the extracellular matrix, angiogenesis, apoptosis, and stimulation of the migration and proliferation of resident progenitor cells [18, 19, 21–29, 31, 104]. All of the studies show amazing prospects for clinical use of UC-MSCs.

## 15. Clinical Studies regarding the Use of UC-MSCs

**Figure 3:**
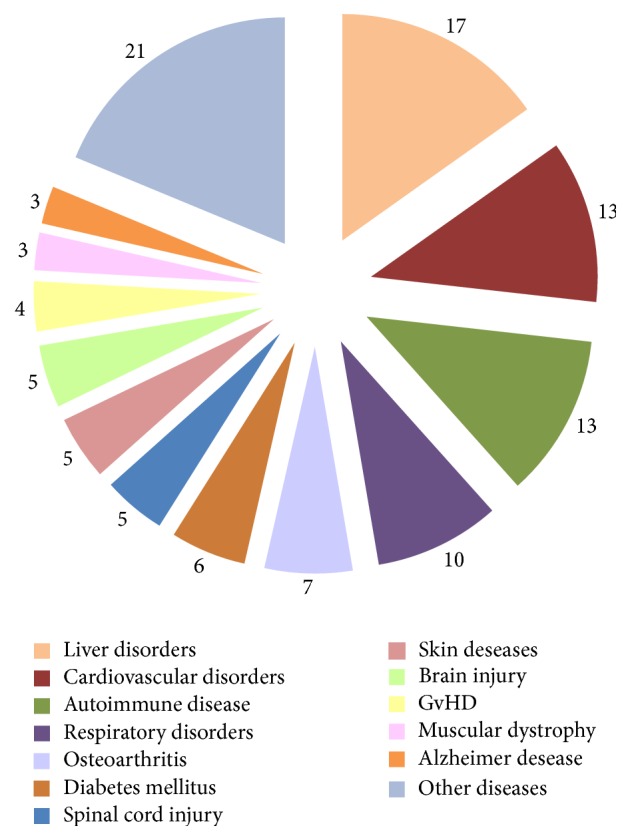
Number of clinical trials for UC-MSCs based therapy (https://ClinicalTrials.gov/).

Currently, the FDA has registered dozens of clinical trials (phases 1–3) on the safety and efficacy of allogeneic unmodified UC-MSCs transplantation for the treatment of socially significant diseases. According to https://www.clinicaltrials.gov/ data [105] (search queries “wharton jelly msc” and “umbilical cord msc”, results that contain “blood-derived” were excluded), UC-MSCs are used for the treatment of acute myocardial infarction, cardiomyopathies, critical limb ischemia, bronchopulmonary dysplasia in infants, HIV infection, diabetes mellitus types I and II, both acute and chronic liver diseases, autoimmune hepatitis, cirrhosis of various etiologies, ulcerative colitis, severe aplastic anemia, Alzheimer's disease, systemic lupus erythematosus, rheumatoid arthritis, myelodysplastic syndrome, hereditary ataxia, spinal cord injury, ankylosing spondylitis, osteoarthritis, multiple sclerosis, Duchenne muscular dystrophy, acute and resistant to steroid therapy “graft versus host” reactions, and other diseases. The diagrammatic representation of clinical applications of UC-MSCs is shown in Figure 3.

At present, the results of only a small part of the clinical studies are published.  Table 6 shows the most promising results of clinical trials (phases 2-3).

In all clinical studies UC-MSCs administration had no side-effects except for several cases of fever. In all clinical trials, only allogeneic transplantation of UC-MSCs is studied. This can be explained quite simply: UC-MSCs banking started a few years ago, so a set of recipient groups for autologous transplantation is not possible for the present. However, there is evidence that the efficacy of autologous and allogeneic MSCs transplantation is comparable [106–108].

The results of clinical trials using UC-MSCs are encouraging, particularly for treatment of autoimmune and endocrine diseases.

## 16. Requirements for the Standardization of Transplant Based on UC-MSCs

The main problem with comparing the results of experimental studies and clinical trials is the lack of a standardized protocol for the isolation, expansion, and cryopreservation of UC-MSCs [42] and of uniform requirements for the final product. The most complete published list of these requirements includes the following items:

1. Tests for virology (HIV-1/2, HBV, HCV, HTLV-1/2, HPV, B-19, CMV, and EBV), syphilis, mycoplasma, and sterility being negative.
2. Phenotype: the percentages of CD73+, CD90+, and CD105+ cells ≥ 98% and the percentages of CD34−, CD45−, HLA-DR−, CD14− or CD11b−, CD79a−, or CD19− ≤ 2%.
3. Viability ≥ 80% after thawing.
4. The content of endotoxin < 2 EU/mL and residual bovine serum albumin < 50 ng/package.
5. No significant upregulation of transcriptase (*hTERT*) gene and oncogenes during large-scale expansion.
6. No significant downregulation of tumor suppressor genes during large-scale expansion.
7. Confirmed potency [109].

## 17. UC-MSCs Are Registered Trademark as UCX®

In the EU, UC-MSCs-based product was registered under the UCX trademark, manufactured by ECBio (Amadora, Portugal). Currently, UCX cells are being used as an active substance for the production of several off-the-shelf biopharmaceutical medicines at the point of initiating clinical trials. Research study for the UCX cells continues toward the use of these cells as an Advanced Therapy Medicinal Product (ATMP) [110].

## 18. UC-MSCs Banking for Clinical Use

Due to the properties demonstrated* in vitro* and* in vivo*, UC-MSCs have attracted the attention not only of the experimental groups but also of clinicians. It is no wonder that biobanks that had specialized previously only in umbilical cord blood storage introduced a new type of service, the storage of cultured MSCs from umbilical cord tissue. Among these biobanks, there are Cryo-Cell International, Inc. (Tampa Bay, USA), Precious Cells BioBank HQ (London, UK), Reliance Life Sciences (Navi Mumbai, India), Thai HealthBaby (Bangkok, Thailand), Cryosite (Granville, Australia), Pokrovsky Stem Cells Bank (Saint-Petersburg, Russia), and other biobanks. The only restriction is that biomaterial must be obtained by Caesarean section; the total number of stored samples exceeds tens of thousands [109]. It is considered that long-term cryopreservation does not change the biological properties of UC-MSCs [111]. From our point of view, the optimal solution in terms of future clinical use is simultaneous banking of cord blood (as a source of hematopoietic stem cells [4]) and cultured MSCs from umbilical cord tissue [112].

## 19. Conclusion

The human umbilical cord is a source of MSCs that have

- a unique combination of prenatal and postnatal MSCs properties;
- no ethical problems with obtaining biomaterial;
- significant proliferative and differentiation potential;
- lack of tumorigenicity;
- karyotype stability;
- high immunomodulatory activity.

Currently isolated and cultured umbilical cord MSCs are a promising storage object of the leading biobanks of the world, and the number of registered clinical trials on their use is currently growing.

## Figures and Tables

**Table 1:** The expression of cell surface markers on UC-MSCs (according to [2, 7–9]).

Positive markers		Contradictory data	Negative markers	
CD10	CD58	CD54	CD3	CD49a
CD13	CD59	CD105	CD11b	CD50
CD29	CD61	CD106	CD14	CD53
CD44	CD73	CD117	CD19	CD56
CD49b	CD90	CD144	CD31	CD71
CD49с	CD106	CD146	CD33	CD80
CD49d	CD166		CD34	CD86
CD49e	CD325		CD38	CD133
CD51	HLA-I		CD40	CD140*α*
CD56			CD45	HLA-II

**Table 2:** The expression of pluripotency markers on UC-MSCs (according to [2, 7–11]).

Positive markers	Contradictory data
REX2	STRO-1
GD2	OCT4
SOX2	SSEA-4
NANOG	
Tra-1-60	
Tra-1-81	
SSEA-1	
DNMT3B	
GABRB3	

**Table 3:** The genes expressed at higher levels in UC-MSCs compared to MSCs derived from adipose tissue, bone marrow, and skin (according to [12]).

Genes	Functions	Expression levels in UC-MSCs compared to		
		BM-MSC	AT-MSC	Skin-MSC
*HAND1* Heart and neural crest derivatives expressed 1	Plays a critical role in heart development	Higher^*∗*^	Higher	Higher

*AFP* Alpha-fetoprotein	The major plasma protein produced in the liver during fetal life	Higher	Higher	Higher

*DKK1* Dickkopf homolog 1	An inhibitor of the WNT-signaling pathway critical for endodermal development	Higher	ns	Higher

*DSG2* Desmoglein 2	An important component of desmosomes in epithelial cell type	Higher	ns	Higher

*KRT8,19* Keratin 8, 19	The major intermediate filament proteins of epithelial cells	Higher	Higher	Higher

*KRT18* Keratin 18	The major intermediate filament proteins of epithelial cells	Higher	ns	Higher

^*∗*^Significantly increased mRNA expression between mutually compared stem cell types (fold change > 2; *P* value < 0.05). ns: not significant.

**Table 4:** Comparison of the differentiation potential of UC-MSCs and MSCs from other sources.

Induction	Parameter	UC-MSCs	BM-MSCs	AT-MSCs	Reference
Osteogenic (35 days)	The average number of bone nodules from one well	19 ± 1.8	7.5 ± 1.3	11 ± 1.7	[13]

Adipogenic (21 days)	The ratio of differentiated adipocytes from the total cells	45 ± 1.5%	39 ± 1%	52 ± 3.2%	[13]

Neuronal (20 days)	The number of primary neurospheres	118 ± 5.2	80.4 ± 3.4	26 ± 3.12	[14]
	The average size of a primary neurosphere	175 ± 2.2 *μ*m	100 ± 3.2 *μ*m	57 ± 0.7 *μ*m	
	The number of secondary neurospheres	47 ± 4.6	7 ± 1.2	Unable to form	
	The percentage of nestin+ cells	91.3 ± 2%	78 ± 1.2%	30.3 ± 6.4%	
	The percentage of *β* III tubulin+ cells	12.5 ± 0.7%	5.6 ± 0.4%	2.4 ± 0.4%	

Neuronal (9 days)	The percentage of *β* III tubulin+ cells	94.6 ± 1.3%	95 ± 1.2%	ND	[15]
	The percentage of cells expressing neuron-specific markers	c. 65%	c. 65%		
	The level of constitutively released dopamine	610 ± 21.7 pg/mL	559 ± 33.5 pg/mL		
	The level of ATP-stimulated release of dopamine	920 ± 45.6 pg/mL	813.5 ± 47.7 pg/mL		

Endothelial (12 days)	Flk-1 expression	17-fold increase	6-fold increase	ND	[16]
	vWF expression	13-fold increase	5-fold increase		
	VE-cadherin expression	16-fold increase	4.5-fold increase		
	Total tubule length of network in Matrigel angiogenesis assay	15 mm	11 mm		

Pancreatic (3 days)	Diameter of formed islet-like cell clusters	Larger (100–200 *μ*m)	Smaller (<100 *μ*m)	ND	[17]
	The percentage of differentiated cells expressing pancreatic-specific marker C-peptide	53.3%	30.9%		
	Insulin secretion on day 1 after differentiation	14 mIU/L	7 mIU/L		

**Table 5:** Preclinical studies regarding the use of UC-MSCs.

Model	Animals	Treatment	Results	Cell fate	Reference
Neonatal lung injury	SCID beige mice	1 × 10^6^ of human UC-MSCs, i.p.	The restoration of normal lung compliance, elastance, and pressure-volume loops (tissue recoil) associated with alveolar septal widening, suggestive of interstitial matrix modification	Cells tended to remain in the peritoneum or retroperitoneum, although eventually some were disseminated to and were retained in the lungs; differentiation into the relevant cells was not found	[18]

Subtotal liver resection (80% organ weight)	Sprague-Dawley rats	1 × 10^6^ of rat UC-MSCs, intrasplenic injection	The stimulation of hepatocyte proliferation and liver weight restoration associated with more rapid recovery of mitochondria number and mitochondrial function of hepatocytes	Cells tended to remain in the spleen, although some part of them migrated to the liver; differentiation into the relevant cells was not studied	[19]

Hindlimb ischemia	BALB/c Slc-nu/nu mice	5 × 10^6^ of human UC-MSCs predifferentiated into endothelial lineage, i.m.	The improvement of blood perfusion associated with increased blood vessel density	Some of transplanted cells were found adjacent to vessel walls; differentiation into the relevant cells was not found	[20]

Streptozotocin-induced diabetes mellitus	BALB/c mice	human UC-MSCs predifferentiated into islets like clusters, 10^3^ clusters in a immunoisolatory capsule, i.p.	The reduction of hyperglycemia associated with increase of body weight	Cells survived and released insulin for 3 months of follow-up before terminating the experiment	[21]

Full skin excision wound	SCID mice	1 × 10^6^ of human UC-MSCs seeded on decellularized amniotic membrane scaffold	The reduction of scar formation with hair growth and improved biomechanical properties of regenerated skin	Cells seeded on decellularized amniotic membrane were grafted onto the area of dermal injury; differentiation into the relevant cells was not studied	[22]

Excisional wound-splinting model	BALB/c nude mice	0,8 × 10^6^ of human UC-MSCs, intradermal injection, 0.2 × 10^6^ of human UC-MSCs, applied to the wound bed	Accelerated wound healing associated with enhancing collagen deposition and angiogenesis	Engrafted cells did not express CD31 or differentiate into the typical cutaneous resident cells	[23]

Myocardial infarction	C57BL/6 mice	2 × 10^5^ of human UC-MSCs, intramyocardial injection	The preservation of cardiac function associated with increased capillary density and decreased apoptosis in the injured tissue	Cells were not found to engraft the murine heart	[24]

Myocardial infarction	New Zealand white rabbits	5 × 10^6^ of human UC-MSCs, subepicardial injection	Improved left ventricular ejection fraction and the percentage of fractional shortening, reduced amount of scar tissue	Some engrafted cells expressed troponin-I, F-actin, and connexin 43	[25]

Myocardial infarction	Guangxi Bama miniswines	40 × 10^6^ of human UC-MSCs, intramyocardial injection	Improved myocardial perfusion and function associated with augmented vessel density and reduced cell apoptosis	Part of the engrafted cells differentiated into cardiomyocytes (cTNT+ cells) and vascular endothelia (vWF+ cells) 6 weeks after transplantation	[26]

Radiation myelopathy	Sprague-Dawley rats	1 × 10^6^ of human UC-MSCs, i.v., 4 transfusions at 1-week interval	Decreased forelimb paralysis and spinal cord histological damage, increased number of neurons in the anterior horn of the spinal cord, the endothelial cell density and the microvessel density in the white matter and gray matter of the spinal cord; increased relative magnitude of spinal cord blood flow; increased anti-inflammatory cytokine expression in the spinal cord	Cell engraftment and differentiation into the relevant cells were not studied	[27]

Dextran sulfate sodium induced acute colitis	NOD.CB_17_-Prkdc^scid^/J mice	2 × 10^6^ of human UC-MSCs, i.v.	The diminution of the severity of colitis and histopathological score associated with decreased myeloperoxidase activity and the expression of cyclooxygenase 2 and iNOS in the colon	Cells were engrafted in the colon; differentiation into the relevant cells was not studied	[28]

Acute carrageenan-induced arthritis and chronic adjuvant induced arthritis models	Wistar rats	1.7 × 10^6^ of human UC-MSCs, intraarticular	Faster remission of local and systemic arthritic manifestations associated with immunosuppression via a repression of T-cell proliferation and TGF-*β*-dependent paracrine promotion of iTreg conversion	Cell engraftment and differentiation into the relevant cells were not studied	[29]

Intracerebral hemorrhage	Sprague-Dawley rats	1 × 10^6^ of human UC-MSCs overexpressing HGF, injection into the left ventricle	Motor function recovery associated with nerve fiber remyelination, reduced myelin-associated glycoprotein activity and higher reactivity in myelin basic protein and growth-associated protein-43	Cell engraftment and differentiation into the relevant cells were not studied	[30]

Sinonasal wound healing	New Zealand white rabbits	6 × 10^6^ of human UC-MSCs overexpressing HGF, i.v.	Improved nasal wound healing recovery associated with reduced collagen deposition and decreased level of the fibrogenic cytokine TGF-*β*1	Cells migrated to the injured mucosa and epithelial layer; differentiation into the relevant cells was not found	[31]

**Table 6:** Clinical studies regarding the use of UC-MSCs.

Disease Number of clinical trials (NCT)	The number of recipients (age) The number of transplanted UC-MSCs The route of administration The frequency of administration	Main results	Reference
Type 1 diabetes mellitus NCT01219465	15 (≤25 years) 2,6 ± 1,2 × 10^6^ i.v. Twice, 4-week interval	(1) During the whole study (24 months), there was no statistical difference between treatment and control groups in mean fasting plasma glucose (FPG) and results of glutamic acid decarboxylase antibody (GADA) test In treatment group compared to control group: (2) Mean postprandial plasma glucose (PPG) levels and glycated hemoglobin HbA1c levels were lower since month 9 (3) Fasting C-peptide levels and mean C-peptide/glucose ratio (CPGR) levels were higher since month 6 (4) The dosage of insulin per day was progressively reduced since month 6. In 8 patients, the daily insulin dosage was reduced by more than 50% of the baseline, and in 3 patients insulin was discontinued. (5) No adverse reactions and no ketoacidosis appeared in treatment group, while in control group ketoacidosis appeared in 3 patients	[32]

Systemic lupus erythematosus NCT01741857	Six (15–60 years) 1 × 10^6^ per 1 kg i.v. Once	One month after transplantation: (1) Serum indoleamine 2,3-dioxygenase (IDO) activity increased (2) Percentages of peripheral blood CD3+CD4+ T-cells decreased	[33]

Systemic lupus erythematosus NCT00698191	16 (17–55 years) 1 × 10^6^ per 1 kg i.v. Once	(1) Significant improvements in the SLEDAI (Systemic Lupus Erythematosus Disease Activity Index) score in all patients 3 months after transplantation and in 2 patients 24 months after transplantation (2) In all patients with lupus nephritis (*n* = 15), proteinuria reduced 3 months after transplantation, 6 months after in 8 patients; 12 months after in 2 patients; 18 months after in 1 patient (3) In 13 patients with hypoproteinemia, serum albumin levels increased (4) In 6 patients with refractory cytopenias, the platelet count increased (5) The percentage of CD4+FoxP3+ T-cells (Treg cells) in peripheral blood increased (6) Serum levels of TGF*β* increased, and serum levels of IL-4 decreased 3 months after transplantation (7) There was no significant difference in IL-10 levels between treatment and comparison groups	[34]

Bronchopulmonary dysplasia NCT01297205	Nine (preterm infants with birth weight of 630–1030 g) 10–20 × 10^6^ per 1 kg Intratracheally Once	(1) There were no significant differences in the duration of intubation between treatment and comparison groups (2) BPD severity was lower in treatment group, regression coefficient 1.7 (3) In treatment group, levels of IL-1*β*, IL-6, IL-8, IL-10, MMP-9, TNF*α*, and TGF*β*1 in tracheal aspirates at day 7 were significantly reduced compared with those at baseline or at day 3 posttransplantation	[35]

HIV-1 NCT01213186	Seven (26–49 years) 0,5 × 10^6^ per 1 kg i.v. Three transfusions at 1-month interval	(1) CD4 T-cell counts and CD4/CD8 ratio increased after 6 months of treatment compared with the individual baseline data as well as with controls (2) No significant alterations in counts of CD3 and CD8 T-cells, CD19+ B cells, CD3−CD56+ NK cells, CD3+CD56+NK T-cells, Lin-1−HLA-DR+CD11c+ mDCs, Lin-1−HLA-DR+CD123+ pDCs, and *γδ*T cells were observed (3) The percentages of naive and central memory T-cells subsets were gradually increased, whereas the effector memory and terminally differentiated effector T-cells subsets were gradually decreased (4) Significantly decreased PD-1 (programmed cell death 1) expression on total CD4, CD8 T-cells, and HIV-1-specific pentamer + CD8 T-cells at months 6, 9, and 12, and significantly increased BTLA (B-lymphocyte attenuator and T-lymphocyte attenuator) expression levels on total CD4 and CD8 T-cells were found at months 9 and 12 (5) Plasma levels of proinflammatory cytokines IFN-*α*2, TNF-*α*, IFN-*γ*, IL-9 (month 6), IL-1ra, IL-12p70, and IL-6 (months 6 and 12), chemokines MIP-1*β*, IP-10, IL-8, MCP-1, and RANTES (months 6 and 12), growth factors PDGF-BB (month 6), and G-CSF and VEGF (months 6 and 12) levels were significantly reduced	[36]

Primary biliary cirrhosis NCT01662973	Seven (33–58 years) 0,5 × 10^6^ per 1 kg i.v. Three transfusions at 4-week interval	(1) There was a significant decrease in serum alkaline phosphatase and *γ*-glutamyltransferase levels at the end of the follow-up period (48 weeks) as compared with baseline (2) No significant changes were observed in serum alanine aminotransferase, aspartate aminotransferase, total bilirubin, albumin, prothrombin time activity, international normalized ratio, or immunoglobulin M levels	[37]

Acute-on-chronic liver failure NCT01218464	24 (24–59 years) 0,5 × 10^6^ per 1 kg i.v. Three transfusions at 4-week interval	(1) The survival rates in patients were significantly increased during the 48-week follow-up period (2) There were increased levels of serum albumin and cholinesterase (12 weeks after the first transfusion), prothrombin activity (1 week after the first transfusion), hemoglobin level, and platelet counts (36 weeks after the first transfusion) (3) Serum total bilirubin (1 week after the first transfusion) and alanine aminotransferase (throughout the 48 weeks of follow-up) levels were significantly decreased	[38]

Myocardial infarction NCT01291329	58 (18–80 years) 6 × 10^6^ Intracoronary infusion Once	(1) The absolute increase in the myocardial viability and perfusion within the infarcted territory was significantly greater than in the placebo group at four months. (2) The absolute increase in the global left ventricular ejection fraction at 18 months was significantly greater than that in the placebo group. (3) The absolute decreases in left ventricular end-systolic volumes and end-diastolic volumes at 18 months were significantly greater than those in the placebo group	[39]

## Abbreviations

MSCs: Mesenchymal stem cells
BM-MSCs: Bone marrow-derived mesenchymal stem cells
UC-MSCs: Umbilical cord-derived mesenchymal stem cells
ISCT: International Society for Cellular Therapy
ESCs: Embryonic stem cells
AT-MSCs: Adipose tissue-derived mesenchymal stem cells.
